# Effect of Shearing and Annealing on the Pasting Properties of Different Starches

**DOI:** 10.3390/gels10060350

**Published:** 2024-05-21

**Authors:** Abdellatif A. Mohamed, Mohamed Saleh Alamri, Hesham Al-Quh, Shahzad Hussain, Mohamed A. Ibraheem, Abdur Rehman, Akram A. Qasem

**Affiliations:** 1 Department of Food Science and Nutrition, King Saud University, Riyadh 11451, Saudi Arabia; msalamri@ksu.edu.sa (M.S.A.); heshamfrnd@gmail.com (H.A.-Q.); mfadol@ksu.edu.sa (M.A.I.); aqasem@ksu.edu.sa (A.A.Q.); 2 School of Food and Biological Engineering, Jiangsu University, Zhenjiang 212013, China

**Keywords:** starch, shearing, pasting, annealing

## Abstract

The functional characteristics of starch can be altered by shear force, which makes the impact on its microstructure of great importance to the food industry. This study investigated the effects of freeze-drying on the gel texture, pasting capabilities, and swelling power of starches made from sweet potatoes (SP), chickpeas (CP), and wheat (WS) combined with Cordia (CG) and Ziziphus gum (ZG). The samples were annealed in water without shearing and in a rapid visco-analyzer (RVA) for 30 min at 60 °C while being spun at 690 rpm. Both native and freeze-dried samples were mixed with 1% or 3% ZG and CG. After annealing, the starches were examined using a texture analyzer and RVA. The results showed that freeze-drying had a substantial (*p* > 0.05) impact on the starch granule, in addition to the effect of annealing. The peak viscosity of freeze-dried native CP and SP starches increased, but the peak viscosity of freeze-dried wheat starch decreased. The setbacks for CP and WS increased, whereas the setbacks for SP varied slightly. Furthermore, it was demonstrated that annealing in an RVA exhibited a substantially lower peak viscosity than annealing in a water bath; the RVA’s shearing effect may have been the cause of this difference. Cordia gum fared better than ZG in terms of peak viscosity, although ZG significantly reduced setback in comparison to CG. Among the various blends, the native WB sample had the lowest hardness (100 ± 4.9 g), while the freeze-dried WB SP sample had the greatest (175.5 ± 4.8 g). Shearing of starches broke up the granules into smaller pieces, which made them gel at lower temperatures. This could be a good thing when they are needed for food uses that require little cooking.

## 1. Introduction

Annealed starches are widely used in the food sector due to their increased degree of heat stability and decreased degree of retrogradation [1,2]. Hydrothermally treated starches can be used in the frozen and canned food industries due to these characteristics. Additionally, beneficial for making noodles are the reduced granular swelling and amylose leaching as well as the increased heat and shear stability that annealing generates [3]. These techniques have also been utilized to raise resistant starch concentrations while preserving granule structure.

Granular starches undergo annealing through incubation in an excessive amount of water for a specific duration at a temperature that is higher than the glass transition temperature but lower than the gelatinization temperature. Previous studies have observed that annealing leads to a rise in the gelatinization temperature and a reduction in the range of gelatinization temperature [4,5,6,7,8]. Additionally, increases in gelatinization enthalpies were detected. According to Knutson [9], interactions between amylose and amylopectin or changes in the coupling forces between crystallites and the amorphous matrix as a result of annealing could be the cause for the rise in enthalpy. The changes in pasting properties as a result of annealing are not the same for all starches and depend on the measuring system (Brabender visco-amylograph vs. RVA), according to DSC results, which showed that, regardless of the type of starch crystal, annealing consistently results in increased endotherm temperature and enthalpy. Moreover, after the annealing procedure, the starches’ granular size grew, but the rates of size variation varied, with larger amylopectin contents leading to faster diameter growth rates and higher final accumulation ratios [10,11]. It should be noted, nevertheless, that annealing is distinct from heat-and-moisture treatments. By restricting the amount of water (35% *w*/*w*) in heat/moisture treatments, molecular mobility at high temperatures is controlled, as well as the gelatinization [12,13]. Low water levels lead to high temperatures, which cause granule physical rearrangement. Results generally indicate that annealing increases the initial temperature of gelatinization and reduces the temperature range of gelatinization. No matter the molecular structure or amylose content of a starch, such alterations are common. Results related to post-annealing gelatinization enthalpies, whether constant or enhanced, can, nevertheless, be conflicting, but it is hypothesized that by increasing double helix registration and optimizing the length of the double helices within crystallites, the annealing process reduces amylopectin crystalline defects [14].

According to the literature, the degree of swelling of the starch granules and their resistance to heat or shear or dissolution will define the hot paste viscosities of starches. On the other hand, the main cause of viscosity growth during the heating of the starch granules is leaching of macromolecules that create an entangled network. Consequently, a number of variables, such as the swelling of the granules and the quantity of solubilized starch, affect the viscosity during pasting. As a result, it can be said that the development of viscosity in starch pastes can be caused by both the production of a dense array of swollen, deformable granules and the leaching of macromolecules [15,16].

The amylose molecules group together as the mixture cools, creating a gel. As a result, the retrogradation tendency of the starch is measured by the viscosity rise upon cooling. One startling finding was the lack of a constant increase in the onset of viscosity development for any starch, which would have been predicted given the steady rise in the annealed starches gelatinization temperature as shown by DSC. Increased viscosity upon cooling was one pasting characteristic shared by all the annealed starches. The amount and molecular weight of the amylose leached from the granules influence the viscosity of the starch gel that forms upon cooling, but it is likely that the remnants of the gelatinized granules also contributed to this viscosity [17]. In fact, a starch gel is composed of a mixture of swollen gelatinized granules, as well as macromolecules that have been leached from the granules [18,19]. It is probable that the remaining granules actually do enhance viscosity because none of the other starches (pea, rice, and potato), with the exception of wheat, demonstrated a rise in amylose leaching after annealing [20,21]. After annealing, the swollen gelatinized granules might become more rigid and shear-resistant, which would increase their viscosity after cooling.

During processing, starch is frequently sheared or homogenized with other components. The structure, pasting, and thermal characteristics might be impacted by the severe mechanical shearing. Shearing impacted the starch granules’ structural integrity, which caused the product’s characteristics to vary during processing [22]. The strength of the granules and the shear pressures used determine how easily they break. Continuous breaking and immediate granule coalescence occur if the impact forces are greater than the granule strength. Granules will not break if the granule strength is greater than the impact pressures. The integrity of native starch granules is extremely susceptible to heating and shear forces, which causes a reduction in the product’s viscosity during the process. Starch granules were broken up and fragmented by shearing, which made them gelatinize at lower temperatures. The viscosities of rice and tapioca starches reduced with increasing shear rates when the shear rate was constant, clearly demonstrating shear thinning properties. However, maize starch behaved like a Newtonian fluid and seemed to be independent of the shear rates [23]. Starch solubility, paste consistency, and paste clarity all rise as a direct result of granule swelling due to heat and shear. Individual granules in a concentrated starch paste gelatinize and expand without restriction until all the available water has been absorbed. The swollen starch granules are more prone to shear breakdown as they swell. All amylopectin double helices have separated at temperatures over the gelatinization onset, but swelling granule structure will be retained until further intense temperatures and shear have been applied. As a result, it is possible to think of the hot starch pastes produced by heat and shear in the presence of water as a mixture of swollen starch granules and granule fragments as well as colloidal and dispersed starch molecules [13]. The goal of this study was to determine how different starches and their mixes with Cordia gum and Ziziphus are affected by the annealing process under shear or no shear circumstances.

## 2. Results and Discussion

### 2.1. Effect of Freeze-Drying on Starch Pasting Properties

**Figure 1:**
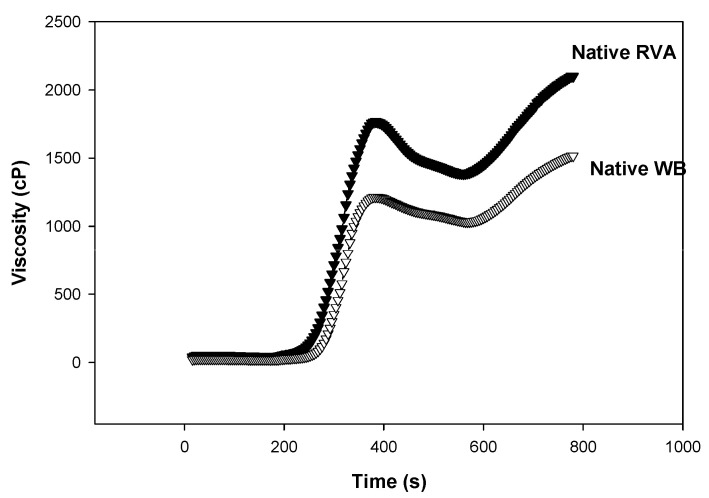
Effect of shearing on the pasting properties of native wheat starch annealed for 30 min at 60 °C in water bath of RVA at 169 rpm.

**Figure 2:**
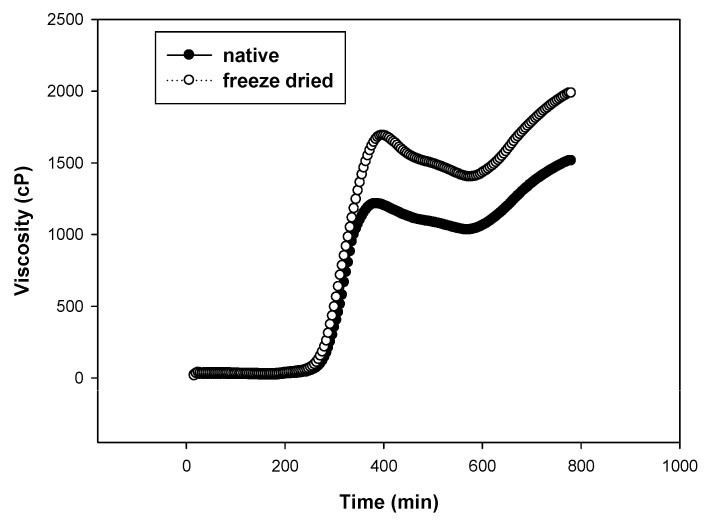
Native and freeze-dried wheat starch annealed in water bath at 60 °C for 30 min.

The investigated starches had amylose contents of 25% for wheat starch (WS), 22.6% for sweet potato starch (SP), and 24% for chickpea starch (CP). The tested (native or freeze-dried) starches were annealed at the same temperature for the same time under shear in the RVA and without shear in the water bath. Table 1 shows how freeze-drying (FD) affected the tested starches’ ability to form a paste. Regardless of the annealing technique (RVA or WB), freeze-drying of sweet potato (SP) and chickpea (CP) starches significantly (*p* > 0.05) decreased the peak (PV) and final viscosity (FV) and increased breakdown (BD), as well as setback (SB), and pasting temperature (PT). On the other hand, by contrasting the samples annealed in RVA to those annealed in the WB at the same temperature and duration, it was possible to determine the impact of shearing on the starches’ pasting properties. It is obvious how shearing decreased the PV of SP and CP starches since it caused the granules to swell more during annealing, which sped up the gelatinization process and decreased PV. The behavior of SP and CP starches was the opposite of that of wheat starch (WS) in RVA, which led to greater PV of both native and FD WS samples. Therefore, we can infer that shearing WS granules at 60 °C for 30 min increased their stability and resistance to shear. This might be a result of the WS’s increased granule packing during annealing, which restricted water absorption, delayed gelatinization, and increased PV. The high amylose content in WS, together with the fact that amylose is found on the surface of the granules, may support the hypothesis that the granules compact. Native and freeze-dried wheat starch both had higher peak viscosities when annealed with shear than when annealed in a water bath (Figure 1), but the freeze-dried WS had higher peak viscosities than the native WS in contrast to SP and CP. Wheat starch displayed swelling after freeze-drying, but it showed severe swelling after annealing in RVA, and it completely gelatinized after annealing in the water bath (Figure 2).The breakdown (BD) is considered another way of looking at the effect of annealing on the native starch pasting after freeze-drying. The BD of freeze-dried wheat starch was significantly higher than that of native starch, and Table 2 lists the differences. WS and SP that had been freeze-dried showed greater breakdown than native starch with or without shear, but CP native starch showed greater breakdown than freeze-dried. The breakdown of the FD and native starches differs in that WS > SP. It is evident how CP starch differs from the other two starches by having a greater native state BD than the other two starches and having a significantly different PV between the native and freeze-dried states. Additionally, the difference in BD, where the compacted granules during annealing appeared to unravel quickly after starting to open up, may be due to the increased amylose concentration of WS.

### 2.2. Effect of Gums on Starch Pasting Properties

Following annealing, Ziziphus gum (ZG) and Cordia gum (CG) both had an impact on the PV of the SP starch. When gums were added, SP starch displayed significantly higher PV than the native or FD, especially when 3% CG was added (Table 1). These are the rankings for the PV of SP starch: 3% CG WB > 3% CG RVA > 1% CG WB > 1% CG RVA. The remaining samples are sorted as follows: NA WB > NA RVA > 1% ZG WB > 1% ZG RVA > FD WB > 3% ZG WB > FD RVA > 3% ZG RVA. The given statistics make it clear that CG was more successful in postponing SP starch gelatinization, which led to a significant increase in PV. While ZG had the opposite effect by encouraging faster swelling, thus reducing the PV compared to CG and the native starch, it is possible that the effect of CG is due to the delay (not suppression) of starch granule swelling, which prolongs the beginning of the gelatinization leading to higher PV. It is also clear that regardless of gum concentration, annealing SP starch in WB continues to produce higher PV than shearing in RVA, which reflects the effect of shearing on starch gelatinization.

Unlike SP starch, while CG increased the PV, CP starch samples with 1% CG exhibited higher PV than the 3%, WB annealing increased the PV more than RVA annealing, and the effect of gums on the PV of chickpea starch (CP) was somewhat similar to SP starch in terms of effect. The PV pattern of CP starch ranking was as follows: 1% CG WB > 1% CG RVA > 3% CG RVA > 3% CG WB. The remaining samples were ranked as follows: NA WB > NA RVA > 1% ZG WB > 3% ZG WB >1% ZG RVA > 3% ZG WB > FD WB > FD RVA. The negative effect of 3% ZG on the PV of CP starch (Table 1) is also noticeable because samples with native starch showed higher PV than samples with 3% ZG, which suggests that higher ZG concentrations may promote starch swelling. In this instance, ZG suppressed the starch PV and helped the granules swell, hastening the gelatinization process. A considerable shearing impact was shown by the fact that WB annealing enhanced PV more than annealing in RVA. Additionally, FD CP starch without gum decreased the PV in comparison to native starch. This may be brought on by granules clustering together and blocking water absorption, which lowers granule swelling.

At 3% CG concentration, wheat starch (WS) exhibited the highest PV compared to 1%. WS behaved differently than SP and CP in the presence of the gum because the PV at 3% ZG was higher than that of CG, as opposed to the other two starches, where CG predominated the PV at 1 or 3% and was followed by ZG (Table 1). Additionally, in contrast to SP and CP, the native WS annealed with shearing showed higher PV than the one annealed in water bath. This different behavior of wheat starch could be attributed to its high amylose content compared to the SP and CP. The native WS annealed in WB and the 3% ZG had the lowest PV. This may be related to the ZG’s inefficient action as a result of its aggregation as well as the overall low PV of WS annealed at WB as compared to RVA. This suggests that shearing is necessary to increase the swelling of the WS granules, while shearing of SP and CP results in reduced PV by accelerating swelling, which then causes a faster rate of gelatinization, resulting in lower PV. According to the literature, some gums increase PV while others decrease it. In contrast to gums like xanthan, guar, locust bean, alginate, and Cordia gum that promoted granule swelling and increased the PV, okra gum was found to reduce the PV of several starches by preventing starch swelling [24,25,26]. The connection between increased viscosity during starch pasting and gum interactions with leached amylose molecules during the initial stages of starch gelatinization may assist in explaining why hydrocolloid has a good impact on PV. Maximum PV determines the starch granules’ capacity to swell freely before breaking down physically; hence, starch with a high maximum PV also has a high swelling power [27]. Some researchers claim that the hydrocolloid is only present in the continuous phase and that as long as the starch granules swell, the concentration of the hydrocolloid in the continuous phase grows, causing a significant increase in the continuous phase’s viscosity [28,29].

In terms of the breakdown (BD), SP and WS had behavior similar to that of the PV in the presence of gum; however, CP displayed a different gum effect on BD. The information in Table 2 demonstrated how the impact of gum on BD was comparable to that on PV because granule swelling directly affects BD and is reflected in PV. As was previously demonstrated, the ranking of CP in the PV was different from the rankings of the other two starches, and this was reflected in the ranking of BD. This made it evident how freeze-drying and annealing, with or without shearing, affected CP starch, which differed from SP and WS starch. This effect was interpreted as the impact of FD on the PV and BD of the starches. This effect was reflected in the effect on PV and BD of CP starch. The data in Table 1 indicate that the gums had a significant impact on the setback (SB) of the starch. ZG tended to lessen the setback by reducing amylose retrogradation, but CG appeared to promote it by permitting maximum amylose retrogradation. The data presented here demonstrate that native SP exhibited the least SB due to the low amylose content, whereas 1% CG raised SP’s SB the most. In terms of CP starch, the SB was highest when 3% CG was present and lowest when 3% ZG was present. The least SB was seen for the native and FD wheat starches, but WS showed the maximum SB at 3% CG and 3% ZG. Since amylose is what causes the SB, it is possible that the differences between the tested starches are caused by the amylose content. WS has the highest amylose concentration; however, its SB comes in third after SP and CP. Remember that the SB shown above is for a gel at 50 °C. CP (1849 cP) had the greatest SB, followed by WS (1730 cP) and SP (1376 cP).

### 2.3. Effect of Gums on Starch Texture (TPA)

The gel texture test was conducted after 24 h of storage at room temperature. Native starch and freeze-dried starch, without gum, were tested for gel hardness. As shown in Table 3, the textural parameters include the following: hardness, which is the force needed to deform the sample; cohesiveness, which is the strength of the internal bonding in the sample; adhesiveness, which is the stickiness of the surface; and springiness, which is the height at which the food can recover between the end of the first bite and the beginning of the second bite. Freeze-drying of SP starch increased the hardness of the gel (Table 2) compared to native starch, which is in line with the reduction in peak viscosity and swelling power of the same starch. This could be attributed to the better amylose network formed in the freeze-dried samples. The addition of the gums reduced the hardness of the freeze-dried samples and increased the hardness of the native SP starch. The data clearly show that gum type and level had a substantial impact on the hardness and adhesiveness of SP gel, but not on the cohesiveness and springiness. The addition of gum caused a noticeable alteration in the CP starch’s hardness, cohesiveness, springiness, and adhesiveness. The hardness, springiness, and adhesiveness of WS, on the other hand, showed significant change as a result of gum type and level, although the cohesiveness showed very minor change. The data showed that the gel hardness of freeze-dried starches was higher than that of the native starch except for CP starch. According to the data in Table 2, shearing CP and WS starches seemed to result in harder gels. However, unlike wheat starch, the gel hardness of SP and CP starches showed no difference between native and freeze-dried. This might be explained by the amylose being distributed throughout the gel as a result of shearing, which made room-temperature amylose networking possible. Freeze-drying had no effect on the gel hardness of SP starch since the gel hardness of sheared and un-sheared gel was the same. Regardless of the amount of added gum or shearing, SP gel hardness in the presence of ZG was higher than CG, while CP starch showed higher gel hardness in the presence of CG. The gel’s hardness appears to be greatly increased by higher gum concentration, which indicates more control over the water in the presence of gum. In comparison to ZG, CG greatly increased the hardness of the wheat starch gel. Higher gum concentrations and after WS shearing both gums produced harder gels. The hardness, springiness, and adhesiveness of WS, on the other hand, showed significant change as a result of gum type and level, although the cohesiveness showed very minor change. Among the various blends, the native WB sample had the lowest hardness (100 ± 4.9 g), while the freeze-dried WB SP sample had the greatest (175.5 ± 4.8 g). This excludes any samples that contain gum. The retrogradation behavior of amylose is directly related to the hardness of gels. A similar pattern to that shown in the hardness values was seen in the setback values of starch blends discovered by RVA studies (Table 1). Gum and starch components are competing for water in the system as a result of the gums’ existence. According to theory, when starches are heated in the presence of gums, the hydrocolloid creates hydrogen bonds with the solubilized starch in swollen starch granules, reinforcing the structure created by the gum and producing high-viscosity paste [10]. The reduction in gel hardness is also reported for the wheat starch sue to annealing process [30]. When the gel is left at room temperature overnight, the gum’s interaction with the amylose and amylopectin in the continuous phase causes the gel to firm up. In general, the starch amylose content and gel hardness have a direct correlation. With the exception of SP starch, freeze-drying had an influence on the setback of the studied starches. At 50 °C, CG-containing samples showed the highest setback and maximal amylose retrogradation. Despite the fact that setback at 50 °C and hardness at room temperature were tested under different conditions, the association between setback and gel hardness was still present. The tight range of amylose content among the studied starches may explain why WS did not display the largest setback and gel hardness, despite having the highest amylose percentage, as well as probable granule shape and compactness.

### 2.4. Swelling Power (SWP)

To provide the right texture, moisture, and water mobility in food systems, hydrocolloids like starches and gums are widely utilized. The swelling, gelatinization, and rheological properties of starch granules can vary as a result of starch–gum interaction in food systems. Table 4 displays the native and annealed starches’ swelling powers (SWP). The results showed that native starch that had been freeze-dried in its native state (without gum) had reduced SWP by 74% for the SP and 73% for the CP but had no effect on SWP for WS. While WS showed an increase in SWP only after annealing in WB and a decrease when annealed under shear, the SWP of SP and CP increased after annealing in water bath or under shearing (RVA). The increase in SWP of the starches annealed in WB can be rated as CP > WS > SP, whereas under shear it was CP > SP > WS. Additionally, the native SP had the highest SWP before annealing, whereas CP had the highest SWP following annealing in WB and WS following annealing under shear. After freeze-drying, the SWP of all three starches showed the highest value after being annealed in a water bath (Table 3). According to these results, freeze-drying without annealing restricted granules swelling, while water bath annealing promoted SWP the most. As a result, freeze-drying decreases the starch’s SWP, which may be due to granules clustering. The amylose content and granule structure of native starch are responsible for its SWP. Wheat starch’s ability to swell was negatively associated with its amylose content. The swelling pattern of the starch granules can be related to the structural type of the amylopectin component. According to a structural examination of amylopectin, starches with stronger swelling powers tend to have higher fractions of longer chains (DP 35) amylopectin [31,32]. The reduction in the swelling power was also reported by Siwatch et al. [33] due to heat moisture treatments. Given these parameters and the high amylose concentration, WS scored highest due to its long chain amylopectin, while CP scored lowest due to its shorter amylopectin chains despite having a lower amylose level than WS. The swelling power, which varies greatly depending on the kind of starch, is the water-holding ability of the swollen starch granules after chilling. It has been claimed that amylopectin can make starch granules expand. The length of the chain and the arrangement of the internal unit chains of amylopectin, water content, the movement of starch molecules in a mixed starch–water system, the movement of water molecules, and the rate at which gelatinized starch crystallizes again are the factors that affect the starch’s ability to gelatinize and retrograde [32,33,34].

Crosslinked starches are most typically utilized in the food industry due to its stable granular structure and limited swelling [35]. According to Ziegler et al. [36], cross-linking decreases the swelling volume of potato and maize starch while improving molecular interactions by covalent bonding. The SWP of wheat starch was significantly increased when xanthan gum (XG) and carboxymethyl cellulose (CMC) were added, according to previous research reports, by maintaining the swollen starch granules close together (cluster). According to Mandala and Bayas [37], in order to slow down water absorption and promote swelling, XG may maintain the granules together while applying more pressure on them. When guar, xanthan, and Arabic gums were added to rice starch, the authors of [38] observed a decrease in the SWP; however, they noticed an increase in the SWP when gellan gum was added. These authors attributed this reduction to the osmotic pressure that was created within the gum’s continuous phase as a result of the starch granules swelling. Additionally, the hydrocolloids prevented the water from penetrating the enlarged granules since their presence increased the viscosity of the continuous phase. Therefore, the literature indicates two effects of gums on the SWP of starch: in one case, an increase in SWP was justified by the gum’s ability to keep granules in clusters, which slows down water absorption and increases swelling. The other situation showed an increase in gum concentration in the liquid phase at the start of granules swelling, which caused osmotic pressure, as well as an increase in viscosity, which prevented water from entering into the granules, leading to a reduction in SWP due to gum addition.

The swelling of starch granules is not immediate and differs between individual granules [39]. According to Crosbie [40], starch granules swelling enables regulated water intake, allowing viscosity to be maintained for a longer period of time until granule rupture, which is followed by a decrease in viscosity (breakdown). Low viscosity, on the other hand, happens when starch granules quickly absorb water, without prolonged swelling, and burst. Since swelling is the initial stage of starch peak viscosity and peak viscosity is a result of granule swelling, it is expected that there will be a significant link between granule swelling and peak viscosity. The swelling volume and power of wheat starch were strongly correlated with the highest viscosity of the starch paste. Consequently, gums that have the ability to progressively absorb water when they start to expand will ultimately enhance the swelling power (SWP) of the starch, and vice versa.

Another mechanism for SWP increase is reported by [41], where gelatinized granules interact adhesively due to xanthan at the beginning of the gelatinization process. It is probable that it can trap them and hold them close. By raising the temperature even further, the continuously leaching amylose and xanthan can insulate the granules and prevent them from expanding any further, causing an increase in the swelling power. Xanthan or sodium alginate increased the swelling power of dry-heated corn starch [42]. Amylose alone inhibits the expansion of starch granules by forming a protective layer around them. Mandala and Palogou [43] have noted another phenomenon where potato starch–xanthan combinations increased the granule swelling by decreasing granule hydration. By looking at the data in Table 4, it is obvious how WB annealing resulted in an increase in SWP of SP and CP starches regardless of gum type or concentration followed by the annealing in RVA (under shear), whereas WS exhibited an un-specified pattern because in some situations, RVA annealing in the presence of both gums had higher SWP, except for the 3% CG. It is clear how the gum affected the SWP of the starches compared to the SWP of native starch (Table 4). With respect to the effect of annealing in WB or RVA on the SWP of the tested starches, the data showed a significant increase in swelling (*p* < 0.05) due to annealing of SP and CP starches in WB followed by RVA annealing. This shows that shearing caused the granules to absorb water more quickly and swell less, whereas annealing in a water bath induced slower absorption and higher SWP, which resulted in enhanced peak viscosity, as mentioned in Section 3. In general, the SWP of RVA-annealed samples and the freeze-dried starches without annealing were comparable. On the other hand, wheat starch displayed a different behavior since, with the exception of the 3% CG mix, the addition of gum enhanced the SWP after annealing in RVA in contrast to SP and CP starch. These findings also revealed no significant difference between the FD WS and WB-annealed samples.

**Figure 3:**
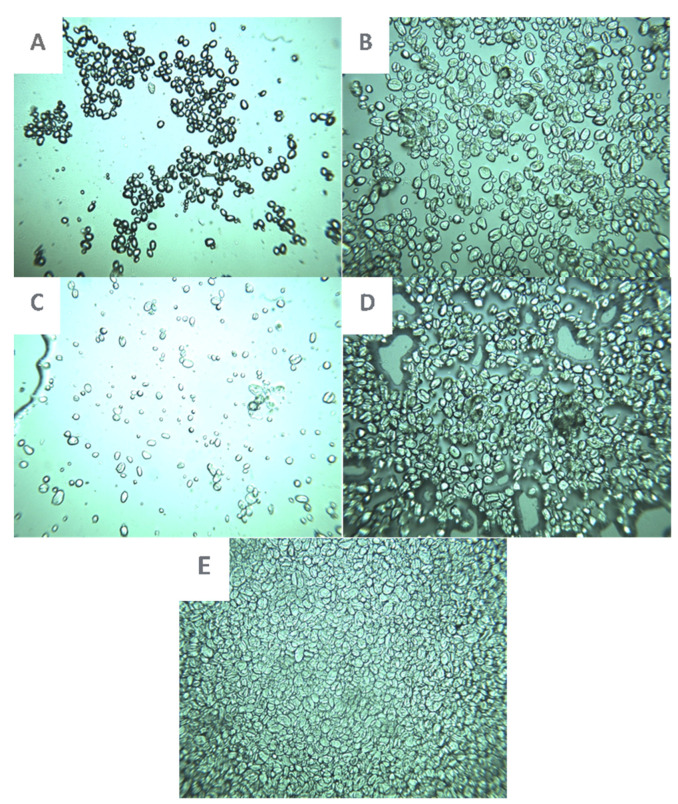
Light microscopic images of starch gels (40× magnification). (**A**) Native chickpea starch; (**B**) Native chickpea starch annealed in water bath; (**C**) Chickpea starch annealed in RVA; (**D**) = Freeze-dried chickpea starch annealed in a water bath; (**E**) Chickpea starch blended 1% Cordia gum annealed in water bath.

**Figure 4:**
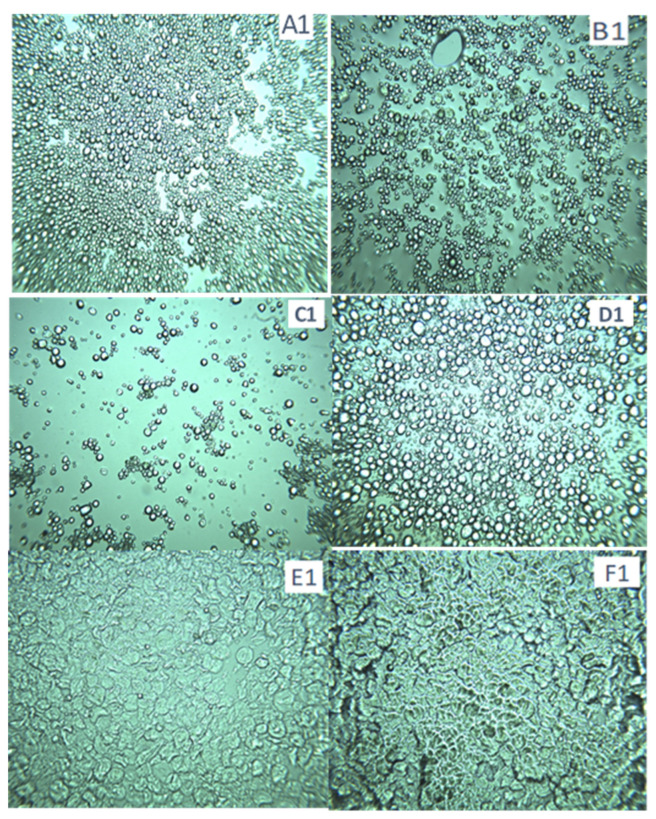
Light microscopic images of starch gels (40× magnification). (**A1**) Native sweet potato starch; (**B1**) Native sweet potato starch annealed in a water bath; (**C1**) Sweet potato starch blended with 3% Cordia gum and annealed in a water bath; (**D1**) Native wheat starch; (**E1**) Native wheat starch annealed in RVA; (**F1**) Native wheat starch annealed in a water bath.

Images of the starches from sweet potatoes (SP) and chickpeas (CP) after various treatments are displayed in Figure 3 and Figure 4. The granules in the native CP starch image seemed smaller than in the annealed samples, while the granules in the native annealed in a water bath displayed swelling. In contrast to samples that were annealed in a water bath and freeze-dried, samples that were annealed in an RVA had dispersed granules and were less swollen. The size of the samples was larger than that of the native sample but less than that of samples that were annealed in a water bath without freeze-drying. Therefore, we can see how freeze-drying can limit granule swelling. This was observed in the data in Table 2, where the swelling power of freeze-dried starches was much lower than native un-freeze-dried. The CP granules were completely enlarged and lacked room in the presence of 1% Cordia. When wheat starch was freeze-dried, it appeared swollen; however, upon annealing in RVA, it exhibited excessive swelling, and upon annealing in the water bath, it became entirely gelatinized (Figure 2). Hence, in contrast to RVA, which is entirely different from CP starch, we can see how sensitive wheat starch is to annealing in the water bath. The swelling power measurements in Table 3 also demonstrated this variation in annealing tolerance. Images of sweet potatoes show that RVA annealing had less of an impact than a water bath, although Cordia gum was responsible for granule clustering.

## 3. Conclusions

Potato, chickpea, and wheat starches have been distinguished based on their physicochemical characteristics or how they are blended with gum under various shear circumstances. This was noted in how freeze-drying affected their gel hardness, swelling power, and pasting properties. The peak viscosity of starches was significantly affected by freeze-drying; chickpea and sweet potato starches showed a considerable decrease in peak viscosity, whereas wheat starch showed an increase in peak viscosity as a result of freeze-drying. While sweet potato starch showed little change on the setback, wheat and chickpea starches showed a substantial rise as a result of the freeze-drying process. In contrast to the other two starches, the effects of freeze-drying on chickpea starch included setback and other post-gelatinization characteristics. This suggests that the effects of freeze-drying extend beyond the granules’ pre-gelatinization properties to include their post-gelatinization parameters. It was also evident that annealing in a water bath considerably raised the peak viscosity in comparison to annealing in an RVA, which may have been caused by the RVA’s shearing action. According to the data, starch blends including Cordia gum exhibited a higher peak viscosity than blends containing Ziziphus gum; however, the setback was considerably reduced in the case of Ziziphus gum. All starches that were annealed in a water bath had a significantly greater swelling power than RVA at the same temperature and duration. The findings of this study can be valuable for the starch processing and other food manufacturing sectors in various food applications that require formulation and process improvement. The scope of the study was only to study these few starches, but it will be further expanded to other commercial starches as well as new sources of starches in our future work.

## 4. Materials and Methods

### 4.1. Materials

Chickpea, sweet potato, and wheat were purchased from a nearby market (Riyadh, Saudi Arabia). Cordia and Ziziphus fruits were procured from a local farm in Riyadh, Saudi Arabia.

### 4.2. Chickpea Starch Isolation

Chickpea (CP) grains were ground and the whole meal was slurried in distilled water (50:50) and mixed using a heavy-duty blender for 5 min (BioloMix, Whirlpool Corporation, Benton Harbor, MI, USA), filtered through a 200-mesh sieve, and the filtrate was centrifuged at 2000× *g* for 15 min (Fisherbrand™ Refrigerated Centrifuge GT2, Hamburg, Germany) [44]. After centrifugation, the top layer of the pellet was removed, and the white pellet was then once again suspended in distilled water and centrifuged as before. A white pure starch fraction was obtained after this procedure was carried out four times. The obtained starch was ground in a coffee grinder, air-dried with acetone, and kept at 40 °C.

### 4.3. Wheat Starch Isolation

Wheat starch was isolated according to [45]. The dough was prepared with wheat flour (flour/water ratio: 2:1, *w*/*w*), placed in a cloth, and thoroughly washed with excess water at 25 °C. The top black layer of the precipitate was removed after centrifuging the filtrate at 2000× *g* for 20 min (Fisherbrand^TM^ Refrigerated Centrifuge GT2, Hamburg, Germany). The white material was then re-suspended in distilled water as before. The isolated starch was powdered in a coffee grinder, air dried, and then kept at 4 °C.

### 4.4. Isolation of Gums

To prevent enzymatic browning, Cordia or Ziziphus fruits were carefully cleaned, and steamed for three minutes. The fruits were blended at a high speed in an auxiliary kitchen mixer (BioloMix, Whirlpool Corporation, Benton Harbor, MI, USA) to create the pulp, which was then centrifuged at 10,000× *g* for 30 min using a Fisherbrand^TM^ Refrigerated centrifuge (GT2) and distilled water added in a 1:3 ratio at 25 °C. After being neutralized, the supernatant was freeze-dried (Alpha 1–4, LD plus, at 0.005 mBarr and 50 °C), ground to powder, sieved through a 60-mesh screen and kept in sealed containers at 4.0 °C for later use. Based on the proportion of the whole fruit, the gum yields for GC and GZ were 1.8% and 11.5%, respectively.

### 4.5. Isolation of Sweet Potato Starch

Sweet potato tubers were carefully washed, peeled, and chopped into small pieces in accordance with [46] procedure. Using an auxiliary kitchen mixer (St 553 Benson Rd, San Antonio, TX, USA), the sliced tubers were blended in distilled water (50:50 *w*/*v*) for three minutes to develop the slurry. The slurry was filtered through a muslin cloth and the overs were re-blended in water, blended, and filtered in the same manner. The top liquid was discarded after allowing the starch in the filtrate to settle for an hour at room temperature. Re-suspending the precipitate in distilled water, it was centrifuged at 2000× *g* for 15 min at 10 °C. The white substance at the bottom of the bottle was reconstituted in distilled water and centrifuged three times to produce pure white starch after centrifugation. The separated starch was dried by air and kept at 5 °C in airtight jars.

### 4.6. Pasting Properties (RVA)

A rapid visco-analyzer (RVA, Newport Scientific, Sydney, Australia) was used to measure the paste-making properties. The starch and combinations with gums or the control (3.5 g at 14% moisture basis) were weighed into designated RVA canisters immediately, and distilled water was then added to make a final weight of 28 g. The slurry was heated to 50 °C for 30 sec, then maintained at 95 °C for 4 min after heating for 4.40 min at 95 °C at a rate of 10.23 °C/min [47]. The sample was cooled to 50 °C in 4 min and maintained there for 2 min. Before reducing to 160 rpm for the duration of the test, the paddle spun at 960 rpm for the first 10 s. The setback, final viscosity, and peak viscosity of the produced gel are included in the profile of the evaluated samples. The data were processed using the Thermocline window software (11.2) from the vendor.

### 4.7. Swelling Power

A simplified swelling power and solubility method based on that proposed by Kusumayanti et al. [48] was used in the experiments. One gram of starch or composite in 50 mL of distilled water was heated at the specified temperature 70 °C for 1 h while gently stirring and then centrifuged at 4500 rpm. for 30 min, and then 0.1 g of the sample was suspended in distilled water (10.0 mL) and was heated in a water bath (Memmert, Mariani, Paola, Greece) at 60 °C for 30 min with intermittent mixing. After heating, the suspension was centrifuged (Alresa Ditacen II) at 16 rpm for 30 min. The sediment was weighted. The (*g*/*g*) *Swelling power* (*SWP*) was calculated as follows:

$$Swelling\;power=\frac{weight\;of\;the\;sedimental\;paste\;\left(g\right)}{weight\;of\;the\;sample\;\left(dry\;basis\right)\left(g\right)}$$

### 4.8. Preparation of Composites

Ziziphus or Cordia gums were used to replace 1% or 3% of the starch in the preparation of a composite made of sweet potato, wheat, and chickpea starches. The composite (50 g) was slurried by adding 90 mL of distilled water and blending it at 550 rpm for 5 min after mixing it by hand for 1 min. The slurry was freeze-dried and preserved for later analysis in jars with tight lids. Native starch (as is), freeze-dried native starch, and composite samples were all studied. Samples were annealed in a water bath without shearing and in a rapid visco-amylograph (RVA) at 690 rpm to investigate the effect of shearing for 30 min at 60 °C. Samples were air-dried after annealing and acetone addition. RVA and the swelling power were used to determine the dry samples’ pasting and swelling properties. The samples were annealed under two conditions, under shear in the RVA and without shear at water bath (WB) at the same temperature and time. Annealing was conducted at the same temperature for the same time.

### 4.9. Texture Profile Analysis of Starch Gels

The texture profile analysis of starch gels was performed according to the method followed by Hussain, Mohamed, Alamri, Ibraheem, Qasem, Shahzad, and Ababtain [28]. RVA experiment starch gels (3 g starch at 14% moisture) were held overnight at room temperature at a height of 35 mm, in 30 mm internal diameter aluminum containers. The Brookfield CT3 Texture Analyzer (Brookfield Engineering Laboratories, Inc. Middleboro, MA, USA), with a 12.7 mm wide and 35 long cylindrical probe, compressed the gels in two penetration cycles at 0.5 mm/s to 10 mm. Gel hardness, springiness, cohesiveness, and adhesiveness were directly measured, whereas chewiness was derived from gumminess and springiness.

### 4.10. Microscopic Examination of Starch Gels

Microscopic images of cooked starch gels were captured on glass slides at a magnification of 40× using a light microscope (Wolfe, Digivu,^TM^, CVM, Carolina Biological Supply Company, located in Burlington, NC, USA).

### 4.11. Statistical Analysis

All experiments were performed in triplicate. Experimental data were analyzed using analysis of variance (ANOVA) and were expressed as mean value ± standard deviation. Duncan’s multiple range test was conducted to assess significant differences among experimental mean values (*p* < 0.05). SAS Foundation 9.2 for Windows was used to analyze the data (SAS Institute, Inc., Cary, NC, USA).

## Figures and Tables

**Table 1:** RVA properties of different starches.

Sample Name	Peak Viscosity (cP)	Breakdown Viscosity (cP)	Final Viscosity (cP)	Setback Viscosity (cP)	Pasting Temp (°C)
	Sweet potato starch (SP)				
FD-SP-RVA	3905 ± 12.3 ^i^	1776 ± 30.12 ^g^	3299 ± 15.12 ^i^	1169 ± 19.10 ^de^	79.20 ± 0.32 ^cd^
FD-SP-WB	4009 ± 13.41 ^h^	1870 ± 30.61 ^f^	3366 ± 15.18 ^h^	1226 ± 13.12 ^c^	78.82 ± 0.23 ^de^
NA-SP-RVA	4433 ± 16.31 ^e^	1882 ± 25.12 ^f^	3713 ± 13.12 ^d^	1162 ± 14.12 ^de^	80.02 ± 0.36 ^b^
NA-SP-WB	4487 ± 11.21 ^e^	1955 ± 20.13 ^e^	3613 ± 14.12 ^e^	1081 ± 19.12 ^f^	79.97 ± 0.42 ^b^
1% CG SP-FD-WB	5407 ± 12.13 ^c^	2418 ± 19.16 ^c^	4365 ± 15.12 ^b^	1376 ± 19.11 ^a^	78.35 ± 0.24 ^e^
1% ZG SP-FD-WB	4240 ± 12.71 ^f^	1917 ± 12.13 ^ef^	3559 ± 16.12 ^f^	1236 ± 12.13 ^c^	79.6 ± 0.31 ^bc^
3% CG SP-FD-WB	6061 ± 12.1 ^a^	2839 ± 30.12 ^a^	4558 ± 15.41 ^a^	1336 ± 11.12 ^ab^	79.15 ± 0.12 ^cd^
3% ZG SP-FD-WB	3929 ± 12.41 ^i^	1638 ± 22.12 ^h^	3486 ± 11.12 ^g^	1194 ± 15.12 ^cd^	80.85 ± 0.12 ^a^
1% CG SP-FD-RVA	5303 ± 12.61 ^d^	2327 ± 14.15 ^d^	4297 ± 13.12 ^c^	1318 ± 13.12 ^b^	78.80 ± 0.14 ^be^
1% ZG SP-FD-RVA	4076 ± 11.41 ^g^	1798 ± 16.12 ^g^	3495 ± 15.12 ^g^	1217 ± 19.12 ^c^	79.62 ± 0.31 ^bc^
3% CG SP-FD-RVA	5927 ± 15.13 ^b^	2708 ± 13.12 ^b^	4532 ± 15.15 ^a^	1313 ± 12.11 ^b^	78.37 ± 0.30 ^e^
3% ZG SP-FD-RVA	3752 ± 12.13 ^j^	1585 ± 15.12 ^h^	3314 ± 12.12 ^i^	1147 ± 11.15 ^e^	80.02 ± 0.12 ^b^
	Chickpea starch (CH)				
FD-CH-RVA	1546 ± 39.10 ^g^	150 ± 12.10 ^fg^	2371 ± 13.12 ^g^	975 ± 15.12 ^def^	78.62 ± 0.36 ^a^
FD-CH-WB	1572 ± 33.12 ^g^	63 ± 3.12 ^g^	2532 ± 10.12 ^f^	1022 ± 21.11 ^de^	77.55 ± 0.42 ^b^
NA-CH-RVA	2137 ± 23.12 ^e^	78 ± 11.10 ^g^	3127 ± 13.16 ^d^	1068 ± 13.21 ^cde^	77.15 ± 0.32 ^b^
NA-CH-WB	2242 ± 21.21 ^d^	57 ± 0.69 ^g^	3268 ± 18.12 ^bc^	1084 ± 15.12 ^cd^	77.10 ± 0.38 ^b^
1% CG CH-FD-WB	3512 ± 35.21 ^a^	968 ± 21.23 ^a^	3400 ± 11.12 ^a^	857 ± 13.21 ^f^	74.65 ± 0.46 ^c^
1% ZG CH-FD-WB	1978 ± 19.21 ^f^	377 ± 12.14 ^d^	2779 ± 12.41 ^e^	1178 ± 14.16 ^c^	77.05 ± 0.23 ^b^
3% CG CH-FD-WB	2308 ± 22.13 ^cd^	785 ± 12.12 ^b^	3372 ± 13.21 ^a^	1849 ± 12.13 ^a^	75.45 ± 0.31 ^c^
3% ZG CH-FD-WB	1946 ± 33.12 ^f^	103 ± 13.11 ^fg^	2552 ± 16.12 ^f^	709 ± 12.13 ^g^	77.50 ± 0.33 ^b^
1% CG CH-FD-RVA	3310 ± 16.21 ^b^	836 ± 15.12 ^b^	3330 ± 13.21 ^ab^	856 ± 11.12 ^f^	74.72 ± 0.35 ^c^
1% ZG CH-FD-RVA	1977 ± 20.12 ^f^	316 ± 13.12 ^de^	2606 ± 13.15 ^f^	947 ± 13.12 ^ef^	77.90 ± 0.39 ^ab^
3% CG CH-FD-RVA	2384 ± 23.12 ^c^	615 ± 16.12 ^c^	3202 ± 12.13 ^cd^	1433 ± 15.12 ^b^	77.05 ± 0.41 ^b^
3% ZG CH-FD-RVA	1897 ± 21.13 ^f^	216 ± 13.12 ^ef^	2407 ± 12.43 ^g^	726 ± 12.18 ^g^	77.57 ± 0.37 ^b^
	Wheat starch (WS)				
FD-WS-RVA	2243 ± 32.1 ^f^	488 ± 14.8 ^d^	2667 ± 33.6 ^e^	911 ± 10.1 ^cd^	87.40 ± 0.91 ^ab^
FD-WS-WB	1703 ± 41.2 ^gh^	313 ± 21.5 ^f^	1950 ± 34.1 ^h^	561 ± 14.2 ^f^	91.875 ± 0.56 ^a^
NA-WS-RVA	1772 ± 19.8 ^h^	381 ± 12.2 ^e^	2103 ± 16.12 ^f^	712 ± 11.2 ^e^	88.125 ± 0.78 ^a^
NA-WS-WB	1190 ± 11.3 ^j^	162 ± 12.41 ^h^	1433 ± 12.1 ^i^	405 ± 20.1 ^g^	93.55 ± 0.65 ^a^
1% CG WS-FD-WB	2462 ± 18.3 ^e^	582 ± 11.2 ^b^	2928 ± 4.61 ^d^	1047 ± 8.9 ^b^	79.52 ± 0.45 ^c^
1% ZG WS-FD-WB	1650 ± 12.1 ^h^	246 ± 15.61 ^g^	2005 ± 3.4 ^fg^	599 ± 5.31 ^f^	93.02 ± 0.92 ^a^
3% CG WS-FD-WB	3080 ± 38.3 ^a^	1072 ± 12.1 ^a^	3738 ± 12.3 ^a^	1730 ± 13.1 ^a^	75.75 ± 0.63 ^c^
3% ZG WS-FD-WB	1553 ± 21.1 ^i^	171 ± 3.6 ^h^	2008 ± 3.4 ^fg^	626 ± 10.1 ^ef^	66.80 ± 0.63 ^d^
1% CG WS-FD-RVA	2732 ± 22.6 ^d^	534 ± 4.5 ^c^	3188 ± 6.12 ^c^	989 ± 11.1 ^bc^	81.47 ± 0.54 ^bc^
1% ZG WS-FD-RVA	2475 ± 24.1 ^e^	540 ± 4.5 ^c^	2813 ± 8.2 ^d^	878 ± 13.20 ^d^	88.55 ± 0.63 ^a^
3% CG WS-FD-RVA	2934 ± 31.5 ^b^	579 ± 3.1 ^b^	3355 ± 3.6 ^b^	999 ± 12.5 ^bc^	81.60 ± 0.82 ^bc^
3% ZG WS-FD-RVA	2836 ± 22.2 ^c^	594 ± 5.7 ^b^	3270 ± 12.1 ^bc^	1013 ± 13.2 ^b^	87.60 ± 0.67 ^a^

NA = native starch; SP = sweet potato: CH = chickpea; WS = wheat starch; cP = centipoise; RVA = rapid visco-analyzer; CG = Cordia gum; ZG = Ziziphus gum; WB = water bath; FD = freeze-dried. Values followed by different letters within each column under a particular starch are significantly different (*p* ≤ 0.05).

**Table 2:** The effect of annealing on the breakdown of sweet potato, chickpea, and wheat starch before and after freeze-drying.

Starch Type	Chickpea		Sweet Potato		Wheat	
	Breakdown		Breakdown		Breakdown	
	RVA ^3^	WB ^4^	RVA	WB	RVA	WB
FD ^1^	1776	1870	150	63	488	331
Native	1882	1955	78	57	381	162
Difference	−106 ^2^	−85	72	6	107	151

^1^ FD = freeze-dried; RVA ^3^ = rapid visco-analyzer; WB ^4^ = water bath; ^2^ The negative sign means that water-bath-annealed chickpea starch has higher breakdown than the RVA-treated chickpea starch, unlike sweet potato and wheat starches.

**Table 3:** Gel texture of sweet potato (SP), chickpea (CP), and wheat (WS) starches using texture analyzer (TPA) after 24 h storage at room temperature.

Sample Name	Hardness (g)	Cohesiveness (g)	Springiness (mm)	Adhesiveness (g)
	Sweet potato starch (SP)			
FD-SP-RVA	170.5 ± 4.8 ^a^	0.490 ± 0.35 ^e^	10.0 ± 0.41 ^b^	0.50 ± 0.06 ^de^
FD-SP-WB	175.5 ± 4.8 ^a^	0.520 ± 0.31 ^cde^	10.20 ± 0.42 ^b^	1.0 ± 0.03 ^a^
NA-SP-RVA	106.5 ± 3.8 ^d^	0.540 ± 0.32 ^cde^	10.25 ± 0.41 ^b^	0.50 ± 0.04 ^de^
NA-SP-WB	100.5 ± 4.9 ^d^	0.640 ± 0.39 ^a^	10.25 ± 0.41 ^b^	0.45 ± 0.06 ^e^
1% CG SP-FD-WB	119.5 ± 4.4 ^c^	0.595 ± 0.33 ^abc^	10.30 ± 0.41 ^b^	0.60 ± 0.06 ^cde^
1% ZG SP-FD-WB	147 ± 5.12 ^b^	0.560 ± 0.31 ^bcde^	10.15 ± 0.42 ^b^	0.90 ± 0.03 ^ab^
3% CG SP-FD-WB	118.5 ± 3.7 ^c^	0.620 ± 0.31 ^ab^	10.15 ± 0.41 ^b^	0.90 ± 0.04 ^ab^
3% ZG SP-FD-WB	143.5 ± 4.1 ^b^	0.525 ± 0.34 ^cde^	11.45 ± 0.44 ^a^	0.65 ± 0.02 ^cd^
1% CG SP- FD-RVA	142 ± 4.1 ^b^	0.560 ± 0.32 ^bcde^	10.15 ± 0.41 ^b^	0.95 ± 0.01 ^a^
1% ZG SP-FD-RVA	152.5 ± 4.6 ^b^	0.535 ± 0.31 ^cde^	10.05 ± 0.41 ^b^	0.75 ± 0.06 ^bc^
3% CG SP-FD-RVA	119.5 ± 4.3 ^c^	0.580 ± 0.33 ^abcd^	10.20 ± 0.41 ^b^	0.85 ± 0.06 ^ab^
3% ZG SP-FD-RVA	146 ± 3.3 ^b^	0.505 ± 0.33 ^de^	9.90 ± 0.41 ^b^	0.70 ± 0.04 ^c^
	Chickpea starch (CH)			
FD-CP-RVA	171 ± 4.9 ^f^	0.62 ± 0.03 ^b^	8.6 ± 0.41 ^c^	0.05 ± 0.12 ^e^
FD-CP-WB	208.5 ± 4.1 ^bc^	0.33 ± 0.03 ^d^	9.05 ± 0.41 ^bc^	0.15 ± 0.12 ^e^
NA-CP-RVA	210 ± 4.3 ^bc^	0.40 ± 0.02 ^d^	8.85 ± 0.48 ^bc^	1.10 ± 0.14 ^ab^
NA-CP-WB	211.5 ± 4.4 ^bc^	0.395 ± 0.04 ^d^	9.65 ± 0.36 ^ab^	1.15 ± 0.12 ^a^
1% CG CP-FD-WB	201.5 ± 3.8 ^cd^	0.355 ± 0.03 ^d^	9.65 ± 0.52 ^ab^	0.95 ± 0.11 ^abc^
1% ZG CP-FD-WB	143.5 ± 3.6 ^g^	0.47 ± 0.01 ^c^	9.45 ± 0.23 ^abc^	0.20 ± 0.12 ^de^
3% CG CP-FD-WB	236.5 ± 4.1 ^a^	0.365 ± 0.02 ^d^	10.10 ± 0.43 ^a^	0.85 ± 0.13 ^bc^
3% ZG CP-FD-WB	183 ± 2.5 ^e^	0.39 ± 0.03 ^d^	8.95 ± 0.41 ^bc^	0.95 ± 0.12 ^abc^
1% CG CP-FD-RVA	192.5 ± 4.2 ^de^	0.47 ± 0.01 ^c^	9.70 ± 0.23 ^ab^	0.15 ± 0.12 ^e^
1% ZG CP-FD-RVA	216.5 ± 3.1 ^b^	0.61 ± 0.03 ^b^	10.05 ± 0.51 ^a^	0.80 ± 0.13 ^c^
3% CG CP-FD-RVA	236 ± 3.6 ^a^	0.39 ± 0.03 ^d^	9.65 ± 0.32 ^ab^	0.45 ± 0.11 ^d^
3% ZG CP-FD-RVA	184 ± 4.2 ^e^	0.79 ± 0.02 ^a^	3.85 ± 0.33 ^d^	0.15 ± 0.12 ^e^
	Wheat starch (WS)			
FD-WS-RVA	136.5 ± 4.61 ^cd^	0.575 ± 0.03 ^c^	9.75 ± 0.4 ^cd^	1.10 ± 0.18 ^a^
FD-WS-WB	126 ± 4.91 ^e^	0.605 ± 0.03 ^c^	10.10 ± 0.4 ^bcd^	0.95 ± 0.18 ^ab^
NA-WS-RVA	114.5 ± 4.41 ^fg^	0.57 ± 0.03 ^c^	10.20 ± 0.4 ^bcd^	0.70 ± 0.17 ^abcd^
NA-WS-WB	105 ± 4.81 ^gh^	0.56 ± 0.03 ^c^	10.25 ± 0.4 ^bc^	1.10 ± 0.18 ^a^
1% CG WS-FD-WB	122.5 ± 4.51 ^ef^	0.595 ± 0.03 ^c^	12.45 ± 0.4 ^a^	0.15 ± 0.13 ^e^
1% ZG WS-FD-WB	104.5 ± 3.8 ^gh^	0.70 ± 0.03 ^b^	9.85 ± 0.4 ^bcd^	0.40 ± 0.12 ^cde^
3% CG WS-FD-WB	154.5 ± 4.92 ^b^	0.56 ± 0.03 ^c^	7.25 ± 0.4 ^e^	0.30 ± 0.16 ^de^
3% ZG WS-FD-WB	100.5 ± 5.32 ^h^	0.74 ± 0.03 ^b^	9.25 ± 0.3 ^d^	0.40 ± 0.14 ^cde^
1% CG WS-FD-RVA	111.5 ± 4.71 ^g^	0.855 ± 0.03 ^a^	10.30 ± 0.3 ^bc^	0.05 ± 0.19 ^e^
1% ZG WS-FD-RVA	142 ± 4.56 ^c^	0.585 ± 0.03 ^c^	10.10 ± 0.4 ^bcd^	0.40 ± 0.18 ^cde^
3% CG WS-FD-RVA	191.5 ± 4.88 ^a^	0.585 ± 0.03 ^c^	10.25 ± 0.4 ^bc^	0.80 ± 0.18 ^abc^
3% ZG WS-FD-RVA	129 ± 4.41 ^de^	0.54 ± 0.03 ^c^	10.75 ± 0.4 ^b^	0.60 ± 0.18 ^bcd^

NA = native starch; SP = sweet potato: CH = chickpea; WS = wheat starch; cP = centipoise; RVA = rapid visco-analyzer; CG = Cordia gum; ZG = Ziziphus gum; WB = water bath; FD = freeze-dried. Values followed by different letters within each column under a particular starch are significantly different (*p* ≤ 0.05).

**Table 4:** Swelling power of sweet potato, chickpea, and wheat starches as is, after annealing in water bath and RVA shearing.

	As is	WB	RVA
Native Sweet Potato	4.37 ± 1.1 ^a^	1.18 ± 0.1 ^b^	1.01 ± 0.7 ^b^
Native Chickpea	2.46 ± 0.8 ^b^	5.09 ± 1.8 ^a^	2.66 ± 1.3 ^b^
Native Wheat Starch	4.49 ± 1.6 ^b^	4.31 ± 1.3 ^b^	6.26 ± 1.2 ^a^
Starch Type	FD	WB	RVA
SP 1% ZG	1.86 ± 0.8 ^a^	1.71 ± 0.8 ^a^	1.01 ± 0.1 ^b^
SP 3% ZG	1.76 ± 0.9 ^b^	2.21 ± 0.1 ^a^	1.05 ± 0.1 ^c^
SP 1% Cordia	1.27 ± 0.23 ^b^	2.28 ± 1.1 ^a^	1.86 ± 0.3 ^b^
SP 3% Cordia	1.25 ± 0.1 ^c^	2.51 ± 0.9 ^a^	2.09 ± 0.8 ^b^
Plain Sweet Potato	1.15 ± 0.3 ^b^	1.43 ± 0.3 ^a^	1.21 ± 0.6 ^b^
Ch 1% ZG	3.38 ± 1.3 ^b^	5.54 ± 1.5 ^a^	3.29 ± 1.4 ^b^
Ch 3% ZG	3.47 ± 1.6 ^b^	6.83 ± 1.9 ^a^	3.49 ± 1.6 ^b^
Ch 1% Cordia Gum	3.03 ± 0.9 ^c^	7.45 ± 1.3 ^a^	3.87 ± 1.3 ^b^
Ch 3% Cordia	4.49 ± 1.4 ^b^	6.70 ± 1.1 ^a^	4.19 ± 1.8 ^b^
Plain Chickpea	0.67 ± 0.1 ^c^	7.95 ± 1.5 ^a^	3.26 ± 1.9 ^b^
WS 1% ZG	3.65 ± 2.1 ^b^	4.36 ± 1.5 ^a^	4.42 ± 1.1 ^a^
WS 3% ZG	4.23 ± 1.5 ^b^	4.27 ± 1.5 ^b^	5.19 ± 2.1 ^a^
WS 1% Cordia	6.09 ± 2.1 ^a^	5.68 ± 1.2 ^b^	6.32 ± 2.3 ^a^
WS 3% Cordia	5.02 ± 1.8 ^a^	5.21 ± 0.8 ^a^	4.42 ± 1.6 ^b^
Plain Wheat Starch	4.48 ± 1.1 ^b^	5.36 ± 0.6 ^a^	4.58 ± 1.4 ^b^

SP = sweet potato: Ch = chickpea; WS = wheat starch; FD = freeze-dried; WB = water bath; CG = Cordia gum; ZG = Ziziphus gum. Values followed by different letters within each row are significantly different (*p* ≤ 0.05).

## Data Availability

The data presented in this study are openly available in article.
